# Simultaneous Bilateral Anterior Shoulder Dislocation After Low-Energy Trauma: A Case Report

**DOI:** 10.7759/cureus.43852

**Published:** 2023-08-21

**Authors:** Diogo Sousa, João Reis, André Guimarães, António Lemos Lopes

**Affiliations:** 1 Orthopaedics and Traumatology, Centro Hospitalar de Trás-Os-Montes e Alto Douro, Vila Real, PRT

**Keywords:** glenohumeral instability, axillary nerve injury, radial nerve injury, elderly trauma, bilateral anterior shoulder dislocation

## Abstract

Unilateral shoulder dislocation is known to be one of the most common joint dislocations. However, simultaneous bilateral shoulder dislocations are rare, especially anterior dislocations. We report a case of an 84-year-old woman who presented to the urgency room with symmetrical bilateral anterior shoulder dislocation 12 hours after falling on a treadmill. She presented with bilateral pain, functional impairment, prominent acromion, flattened shoulder, and, in the right upper limb, paresthesias on the dorsum of the hand and extension deficit of the fingers. Closed reduction of both shoulders was performed under sedation, and she was immobilized bilaterally with an arm sling in internal rotation. A full recovery was achieved six months after the injury.

## Introduction

Shoulder dislocation is recognized as the most common body dislocation (85% of cases). However, bilateral shoulder dislocations are rare and usually posterior due to violent and involuntary muscular contractions (epilepsy or electric shock). Bilateral symmetrical anterior shoulder dislocations (BSASD) are even less common with only a few cases reported in patients above 40 years old [1].

## Case presentation

**Figure 1:**
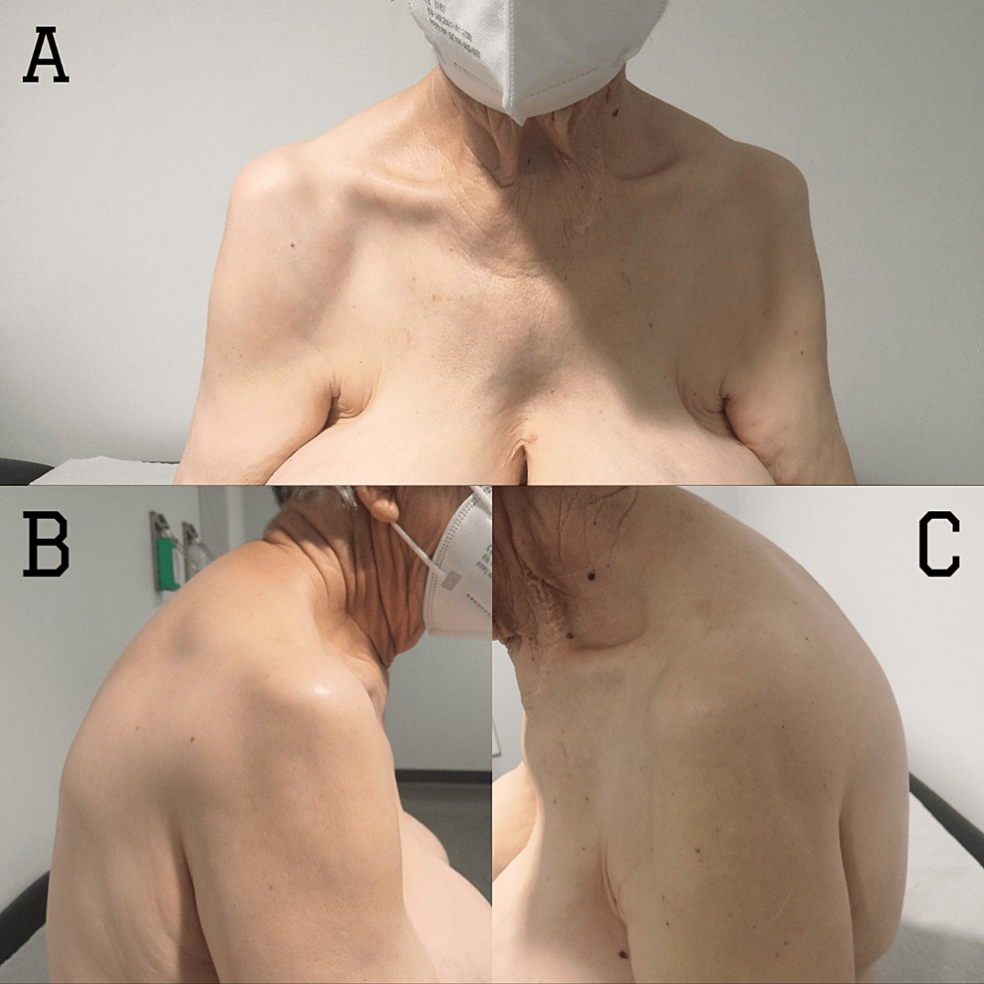
Clinical aspect of shoulders of bilateral symmetric anterior shoulder dislocation on the front (A), right-side (B), and left-side (C) views

An 84-year-old woman fell on a treadmill and kept her grip on the handrail with both hands. This mechanism led to a symmetric and synchronous anterior flexion, abduction, and external rotation of both shoulders. She presented to the emergency service 12 hours after the injury, presenting with bilateral shoulder pain, functional impairment, prominent acromion, and flattened shoulder (Figure 1). In the right upper limb, she had an injury to the axillary nerve (badge sign) and radial nerve (paresthesias on the dorsum of the hand and extension deficit of the fingers). No other neurological or vascular injuries were identified prior to the reduction. As per previous medical history, she had mild dementia but was still autonomous in daily life activities. She had no past surgical history in both shoulders and did not complain of previous pain or functional impairment.

**Figure 2:**
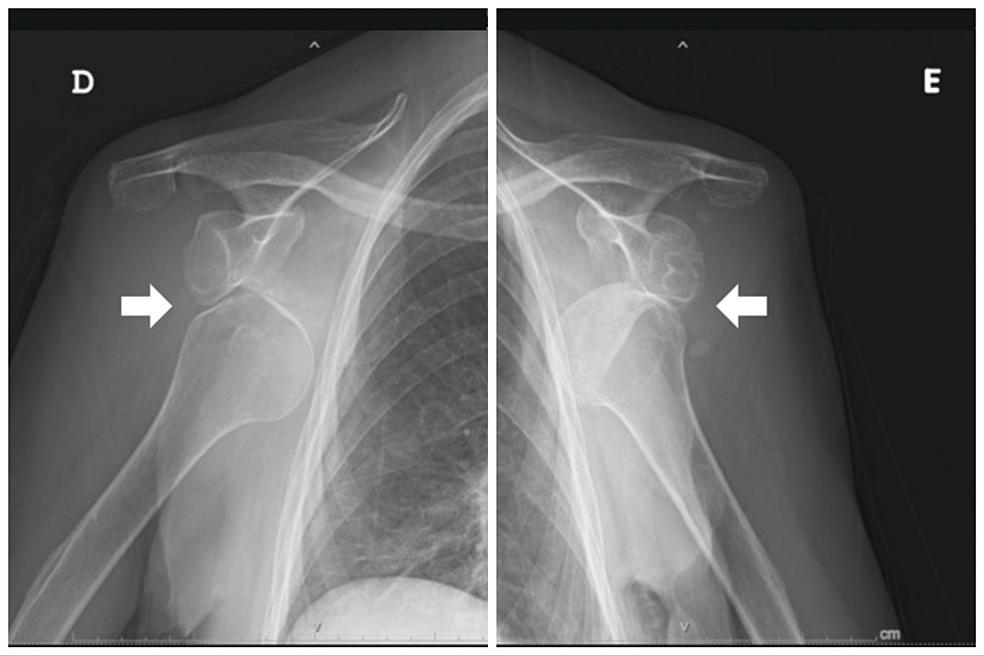
Shoulder radiographs of bilateral symmetric anterior shoulder dislocation (arrows) on the right (D) and left (E) sides (anteroposterior view)

The radiologic evaluation showed a bilateral symmetrical anterior shoulder dislocation (Figure 2).

**Figure 3:**
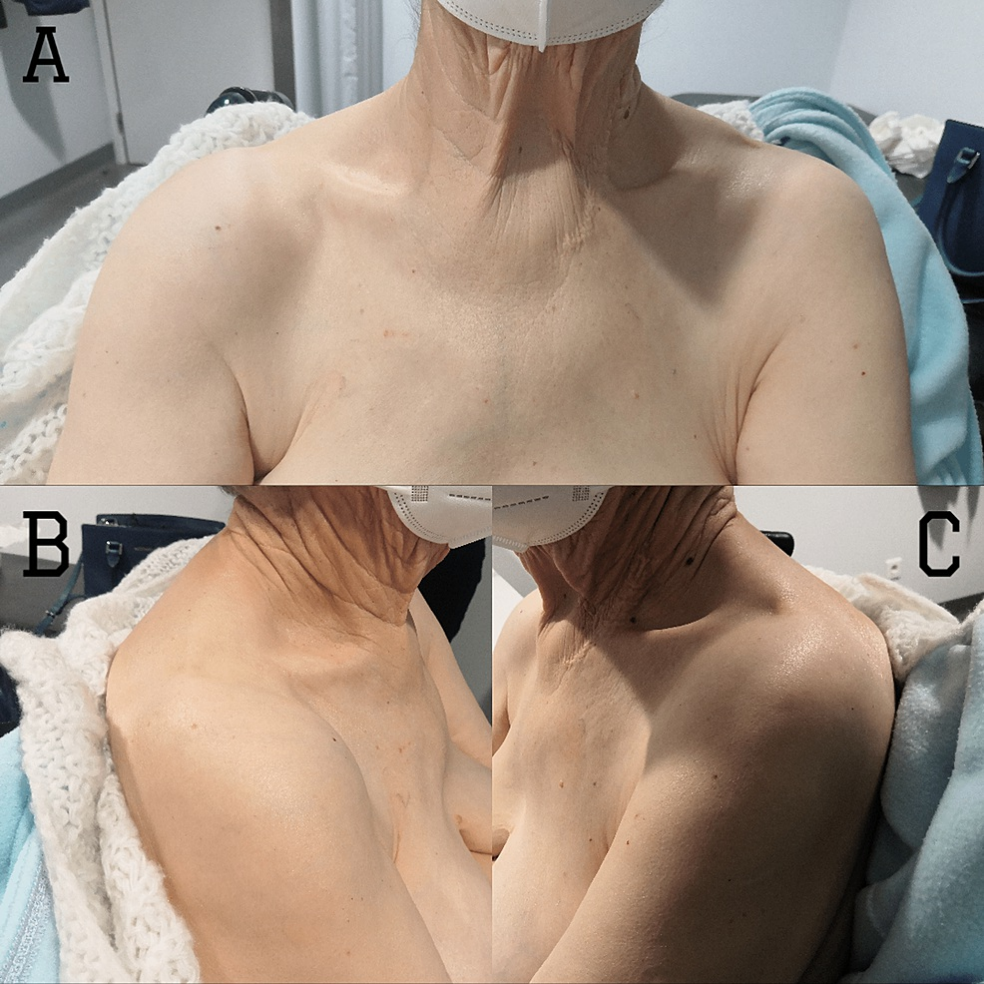
Clinical aspect of shoulders after reduction of bilateral symmetric anterior shoulder dislocation on the front (A), right-side (B), and left-side (C) views

**Figure 4:**
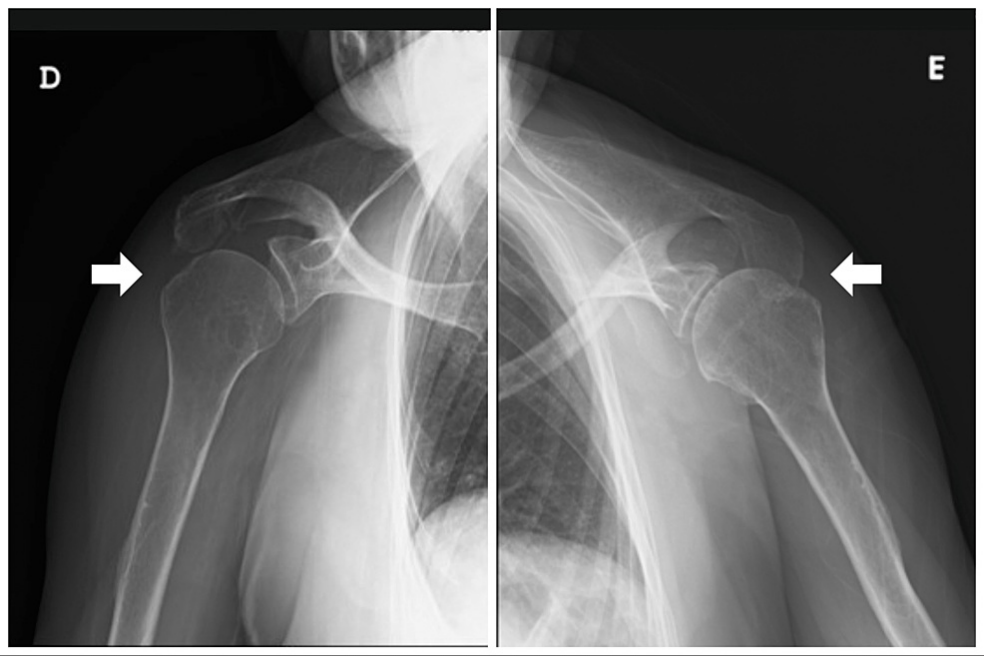
Shoulder radiographs after reduction of bilateral symmetric anterior shoulder dislocation on the right (D) and left (E) sides (anteroposterior view)

Closed reduction of both shoulders was performed under sedation (Figures 3, 4). The left shoulder was reduced with the Kocher maneuver, and the right shoulder was reduced with traction-countertraction technique after the failure of the Kocher maneuver. After reduction, she maintained the previously described deficits and also presented with weakness in active elbow extension and shoulder abduction, on the right side. She was immobilized bilaterally with an arm sling in internal rotation.

After 15 days, she presented with a nearly asymptomatic left shoulder and was allowed mobility according to tolerance, restricting only extreme abduction and external rotation. Regarding the right upper arm, she recovered from radial nerve injury but remained with shoulder pain and axillary nerve injury, so immobilization was indicated for more than 15 days.

An electromyography of the right upper limb was performed approximately one month after the traumatic event, which showed severe but incomplete axonal injury of the axillary nerve, with signs of active denervation and no voluntary muscle activity or signs of ongoing reinnervation.

Conservative treatment was chosen, including physical therapy, and she had a full recovery after six months, with no persisting pain or neurological deficits.

## Discussion

Shoulder joint dislocation, also called glenohumeral joint dislocation, is one of the most commonly encountered cases in an emergency setting. However, simultaneous bilateral shoulder dislocations are fairly uncommon, posterior bilateral dislocation being more frequent than anterior due to violent and involuntary muscle contractions (electrical shock, seizures, or hypoglycemic episodes) [1]. Contrarily, BSASD is more related to trauma (most often high-energy trauma, such as highspeed accidents) and is mainly seen combined with a fracture [1,2]. Dislocations are considered acute or recent when recognized within 21 days from the trauma and chronic or old if recognized after 21 days.

The diagnosis of BSASD may easily be missed or delayed due to its rarity and the fallacious symmetry of the shoulder girdle during clinical examination. In fact, more than 10% of all bilateral shoulder dislocations are not diagnosed immediately [3]. Then, the clinical examination must be rigorous and systematically complemented by x-rays.

Various mechanisms may contribute to BSASD. In this case report, the traction mechanism took a main role. This happened because a traction force was applied superiorly or anteriorly through the extremity with shoulders in FABER (Flexion, ABdution, and External Rotation) [4,5]. Other described mechanisms are indirect force, indirect lever, direct force, and complex mechanisms [6]. Nevertheless, despite being very rare, there are also described cases of nontraumatic bilateral symmetrical anterior shoulder dislocation related to inflammatory diseases, likely due to substantial capsular and ligamentous destruction [7].

BSASD is associated with complications in approximately half of the cases. The most common associated complications are fractures, mainly of the greater tuberosity of the proximal humerus [6]. Neurovascular injuries are also related to BSASD and, despite being rare, should be promptly looked for, especially with traction mechanism lesions, since they put the highest risk on the neurovascular structures. The posterior and medial cords of the brachial plexus are often compressed [6]. Nevertheless, sensorimotor function usually recovers with a good outcome if promptly recognized and addressed. Other reported associated injuries are rotator cuff tears, the interposition of the long biceps tendon in complex proximal humerus fractures, and osseous Bankart lesions [6].

## Conclusions

Bilateral shoulder dislocation is usually posterior and is seen in the context of a violent muscle contraction (epilepsy, electric shock, and polytrauma) or in the context of hyperlaxity or neuromuscular disease. BSASD is an extremely rare entity, with only a few cases described in the literature, requiring a symmetric and synchronous traumatic lesional mechanism. Due to its rarity and associated fallacious clinical presentation, the clinical examination must be rigorous and systematically complemented by x-rays to allow proper diagnosis, treatment, and follow-up.
